# Land Use/Cover Dynamics in Response to Changes in Environmental and Socio-Political Forces in the Upper Reaches of the Yangtze River, China

**DOI:** 10.3390/s8128104

**Published:** 2008-12-09

**Authors:** Xing Wu, Zhenyao Shen, Ruimin Liu, Xiaowen Ding

**Affiliations:** 1 State Key Laboratory of Water Environment Simulation, School of Environment, Beijing Normal University, Beijing 100875, P.R. China; E-Mails: yuntian112@gmail.com; liurm@bnu.edu.cn; 2 Energy and Environmental Research Center, North China Electric Power University, Beijing 102206, P.R. China; E-mail: binger2000dxw@163.com

**Keywords:** Land use/cover change, Driving forces, Environmental condition, Socio-political factor, Upper reaches of the Yangtze River, China

## Abstract

Land use/cover change (LUCC), which results from the complex interaction of social, ecological and geophysical processes, is a major issue and the main cause of global environmental change. This study analyzed the land use/cover dynamics and their environmental and socio-political forces in the upper reaches of the Yangtze River from 1980 to 2000 by using remote sensing, climatic and socio-economic data from both research institutes and government departments. The results indicated that there had been significant land use/cover changes between 1980 and 2000 in the study area, which were characterized by a severe replacement of cropland and woodland with grassland and built-up land. The transition matrices highlight the dominant dynamic events and the internal conversions between land use/cover types during the study period and reveal two distinct transition phases. Land use/cover changes in the upper reaches of the Yangtze River during 1980 to 2000, while restricted by environmental attributes, were strongly driven by socio-political factors. However, excessively pursuing higher land use benefits likely results in serious environmental degradation. This study suggests that the restructuring of land use should be based on land suitability and sustainable protection of fragile environment in the upper reaches of the Yangtze River. A thorough comprehension of historical changes will enhance our capability to predict future land use change and contribute to effective management strategies and policies for the rational land use.

## 1. Introduction

Land use/cover change (LUCC), which results from the complex interaction of social, ecological and geophysical processes [1], is a major issue and the main cause of global environmental change [2-3]. Fast and drastic land use/cover changes during the past decades due to urban and agricultural developments have been reported around the world [4-9]. Recently, issues related to LUCC have attracted interest among a wide variety of researchers, ranging from those who try to model spatial and temporal patterns of land conversion to those who want to understand the causes and influences of LUCC [10-15]. Although climate change, urbanization and population growth have been considered the most common factors contributing to LUCC on a global scale, there is no consensus concerning the relationships between LUCC and its driving forces, largely because of the complex interactions among physical, biological, economic, political and social factors. Therefore, to fully understand the process of LUCC, it is equally important to look into the environmental, socio-economic and institutional backdrop against which the LUCC have taken place.

There are land use/cover changes studies revealing close relationships to environmental attributes [16-17], whereas other studies show a better correlation between LUCC and socio-economic characteristics [10, 18]. The relationship between land use/cover and environment may be weakened by human activities reducing the constraints of environmental factors [17, 19]. Furthermore, Verburg and Veldkamp have stated that a single research approach does not sufficient for a complete analysis of land use change [20]. Instead, a combination of multiple approaches is much more necessary for LUCC research [21-22].

Understanding the process, dynamic and driving forces of LUCC implies an integrating technique involving multiple disciplines. Although there are various methods that can be used in the collection, analysis and presentation of resource data, remote sensing (RS) and geographic information system (GIS) technologies have been recognized as powerful and effective tools and are widely applied in detecting the spatio-temporal dynamics of LUCC [2, 4, 23-24]. Repeated satellite images and/or aerial photographs are useful for both visual assessments of natural resource dynamics occurring at a particular time and space as well as quantitative evaluation of LUCC over time [25]. Analysis and presentation of such data, on the other hand, can be greatly facilitated through the use of GIS technology [6, 11]. In addition, there are widely used approaches for detection of changes and statistical analysis which allow, on the one hand, identification of structural variation among different land cover patterns and on the other hand, diagnosis of land use changes based on climate and socio-economic data time series [18, 26-28]. Therefore, these LUCC time series analyses and the identification of driving forces responsible for these changes are significant, not only for the sustainable management of land resources and regional development, but also for the projection of future land use trajectories [22, 29-30].

In the past two decades, with the rapid growth rates of economy, land use patterns have also been dramatically changed in China. It has been well documented that obvious land use change, especially with regard to urban sprawl and loss of cultivated land, has occurred in the processes of environmental changes, industrialization and urbanization in the whole country [2, 31-32]. Regional land use changes in the eastern and coastal regions of China have received great attention [12, 33-34]. Comparatively, land use changes in its western undeveloped regions have attracted much less attention [11, 35], especially in the upper reaches of the Yangtze River, which represents a very fragile ecosystem threatened by increasingly intensive and unsustainable land-use practices, such as reclaiming land by destroying forests and farming on steep slopes [36]. As a result of the typical climatic changes and human activities, the LUCC in this region is quite different from that in lowlands at the same latitudes. Most parts of the upper reaches of the Yangtze River are problematic and as they are geologically unstable with large areas under greater inclinations. Because of the population growth and unequal land distribution forcing farmers to expand agriculture on marginal lands on steep slopes, severe soil erosion has caused many disasters, such as landslide and flood, and in turn leading to serious eco-environmental degradation in this region.

The objectives of this study were: (1) to identify the land use/cover dynamics in the upper reaches of the Yangtze River during the periods from 1980 to 1990, and 1990 to 2000 by using multi-temporal remotely sensed data and GIS; (2) to discuss the major driving forces for such changes based on the environmental attributes and socio-political factors; and (3) to develop alternative concepts and strategies for more effective nature conservation and land use planning.

## 2. Study area

**Figure 1.**
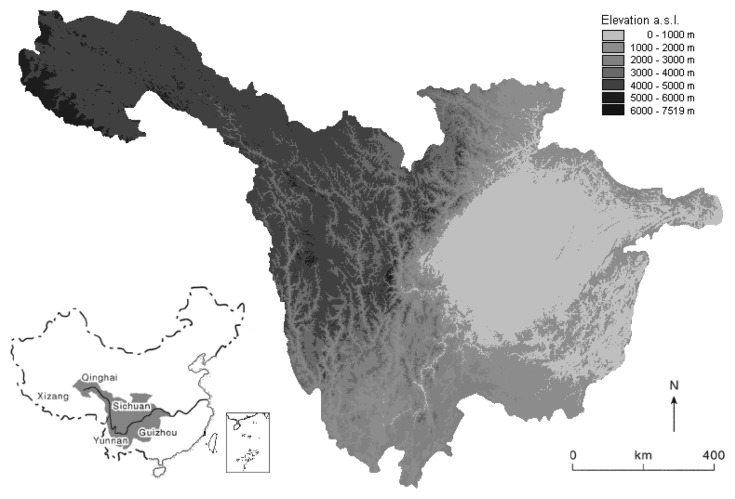
Location and topographical situation of the study area.

The site of this study, the upper reaches of the Yangtze River (90°30′–112°04′ E and 24°50′–35°35′ N), is situated in the southwestern part of China (Figure 1). The watershed covers a region from Yichang of Hubei Province to the river's origin. The total area is about 1,000,000 km^2^ with a total population of more than 160 million, which accounts for 58.9% of the area and 35% of the population in the whole Yangtze valley, respectively [37-38]. Rising on the arid Qinghai-Tibet plateau and descending into the Sichuan Basin, the upper Yangtze basin contains about 31.3% of land over 4,000 m and includes a diverse range of environments [39-40]. The climate in the upper Yangtze is mainly controlled by elevation, due to the effect of Qinghai-Tibet plateau on the atmospheric circulation [41]. The plateau constrains the penetration of the monsoon, resulting in the complex pattern of precipitation within the basin, ranging from arid in the extreme northwest (<250 mm/year) to subtropical monsoon climates in the east (>1,000 mm/year). Population densities are similar varied ranging from <10 people/km^2^ in the mountainous west to >500 people/ km^2^ in the Sichuan Basin, which is one of the most populous areas of China [42].

Within this area, 90% or more of the land is mountains and plateaus, in which the ecosystem is very fragile and subject to soil erosion due to inappropriate use of land. This includes land-use problems such as intensive and widespread deforestation and reclamation, farming on steep slopes and overgrazing, which led to server eco-environmental degradation [43]. Such degradation not only undermines the sustainability of on-site land use, but also causes off-site problems related to the ecological security of the lower reaches of the river. At present, the situation of economic development in the upper reaches is far inferior to that of the middle and lower reaches of the Yangtze River, and is characterized by a simple subsistence economy, mainly for self-consumption in most hillside regions [31]. However, the development of the watershed is not uniform. Sichuan basin, with relatively flat landform, is one of the most fertile and economically important areas in the upper reaches of the Yangtze River, where most of the commercial activities are concentrated. The local economy and employment opportunities in this region differ from rural areas, where people are primarily dependent on agriculture, deforestation and livestock raising for their livelihood. Therefore, the high variability in the ecological and economic conditions makes the watershed an appropriate site to study the dynamics of land use and the factors associated with it.

## 3. Methodology

### 3.1. Data

The analysis of LUCC in the upper reaches of the Yangtze River is based on three land use/cover maps at a 1:100,000 scale. These maps were derived from historical Landsat TM (Thematic Mapper) satellite images, which were acquired in 1980, 1990, and 2000, respectively, by Institute of Geographic Science and Natural Resources Research of the Chinese Academy of Sciences [6, 23, 32, 44]. The dataset is based on the remotely sensed data for the whole country with a maximal spatial resolution of 30 m. The Landsat images were enhanced using the linear contrast stretching and histogram equalization to help identify ground control points in the rectification to a common ALBERS coordinate system based on 1:100,000 topographic maps of China. An efficient classification system was drafted and an effective research team was organized to work on this dataset through human-machine interactive interpretation to guarantee classification consistency and accuracy. After geometrical image correction and geo-referencing, the average location errors were estimated as less than 50 m (about 2 pixels). A out-door survey and random sample check (covering a line survey of 70,000 km and 13,300 patches) verified that the average interpretation accuracies for land use/cover were 92.92% and 97.45% for land use change interpretation [23, 32].

**Figure 2.**
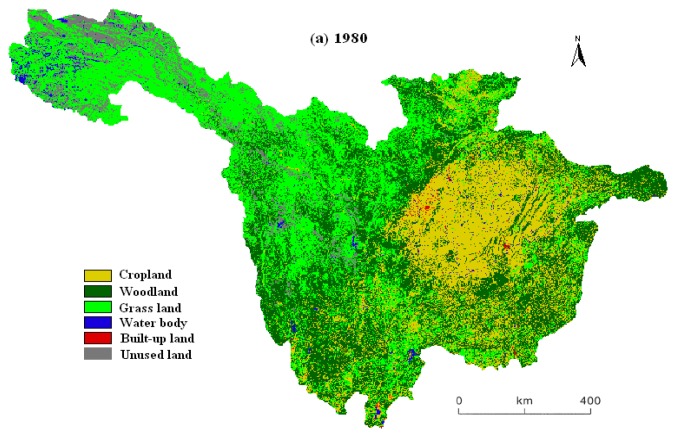

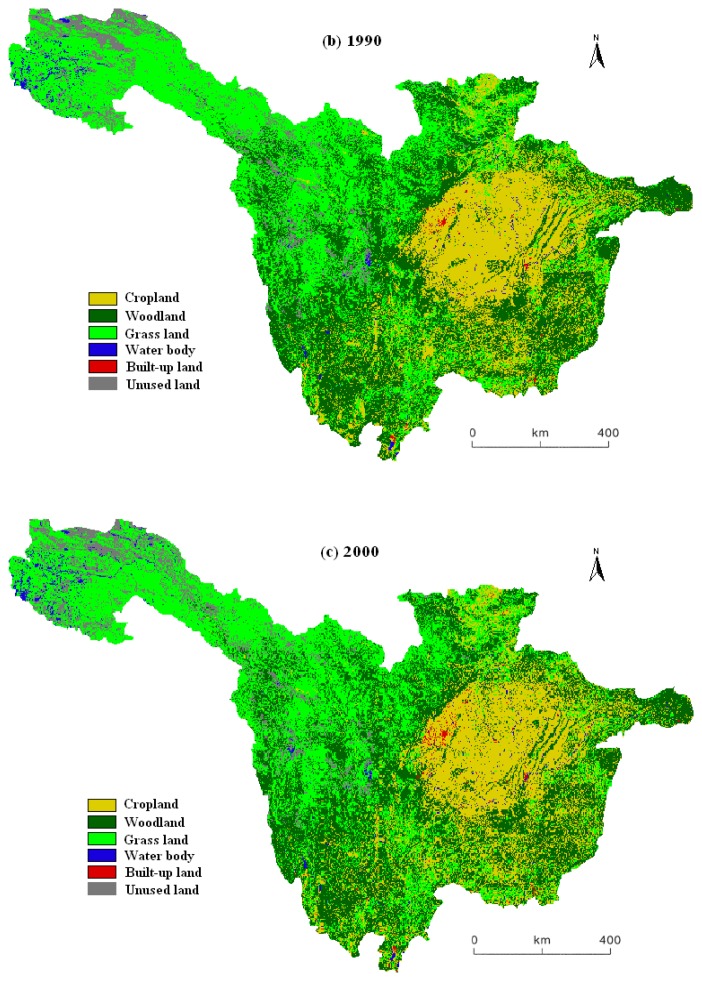
Land use in the upper reaches of the Yangtze River in 1980 (top), 1990 (middle) and 2000 (bottom).

A hierarchical classification system of 25 land use/cover subclasses was applied to the Landsat TM data. The 25 subclasses were further grouped into six aggregated classes: cropland, woodland, grassland, water body, unused land and built-up land. Cropland includes paddy and dry farming land; Woodland includes forest, shrub and others; Grassland includes three density-dependent types: dense, moderate and sparse grass; Water body includes stream and river, lake, reservoir and pond, glacier and firn, beach and shore, and bottomland (overflow land); Unused land includes sandy land, Gobi, salinized land, wetland, bare soil, bare rock and others such as alpine desert and tundra; Built-up land includes urban area, rural settlements and others such as roads and airports [6, 23, 32]. In this study, we measured the variations among the six aggregated land use/cover types by converting the three vector format maps into raster format with a spatial resolution of 1 km × 1 km using ESRI's ArcView 3.3 spatial analysis module. Figure 2 illustrates the three gridded and classified land use/cover maps in 1980, 1990 and 2000 in the upper reaches of the Yangtze River.

In order to reveal the main driving forces for LUCC, we selected some related data reflecting environmental changes and human activities. The long-term climatic information in our study area, including annual time-series dataset of temperature and precipitation from 106 weather stations during 1960-2000, was obtained from the Meteorological Information Center, China Meteorological Administration. To characterize the study area with respect to the most important physical conditions, the elevation and slope inclination were selected as the principal limiting parameters for land use capacity [49-51]. These physical constraints were identified in several studies to be related to LUCC [7, 17, 43]. Elevation data were derived from a digital elevation model (DEM) at a 1:250,000 scale, which was obtained from the State Bureau of Surveying and Mapping of China. Slope inclination was also calculated from the DEM by utilizing the slope function in the ArcView 3.3 spatial analysis tool, which calculates the maximum rate of change of elevation between each cell and its eight neighboring cells. According to local conditions of agriculture production, the elevation was classified in to 4 levels: <1,000 m, 1,000-3,500 m, 3,500-5,000 m and >5,000 m, the slope degree was also classified into 4 levels: 0-5, 5-10, 10-25 and >25 degrees. In addition, some socio-economic data about population and gross domestic product (GDP) were collected from the local governments and used to analyze potential driving forces resulting in LUCC in the upper reaches of the Yangtze River [45]. Furthermore, historical information about institutions and natural resource policies related to the upper reaches of the Yangtze River over the past decades was obtained to provide a context from which to analyze their impact on land use/cover in this region.

### 3.2. Methods

In addition to analyzing the changes in the amount of land use types, the temporal transitions among the types are also important as they can express the detailed information of the conversion between different land use types. Thus, we can analyze the transition matrix of land use types for different periods, to exactly identify the process of land use change. Based on analysis of remote sensing images, a post-classification comparison change detection algorithm was used to determine the internal variations of land use/cover in upper reaches of the Yangtze River between two different periods: from 1980 to 1990, and from 1990 to 2000. The land use polygon themes for 1980, 1990 and 2000 were overlaid two at a time in ArcView GIS and the area, converted from each of the classes to any of the other classes, was computed. For each land use/cover category *i* in a transition matrix *A*, the change between the two periods was calculated according to the following equation (1) [11, 22].

(1) $$CH_{i}=(p_{i.}-p_{.i})/p_{.i}\times 100$$

where:
*CH_i_*is the change of land use/cover in row *i* relative to the previous compared year;*p_i._*is the row total of grid cells for category *i*;*p_.i_*is the column total of grid cells for category *i.*

The magnitude and direction of LUCC were determined based on the transition matrix. For exploring the internal conversion between different land use/cover types, which took place between the two compared periods, we treated the change (increase or decrease) of a type of land use/cover in a given year relative to the compared year as a result of several “gain or loss” conversions. Therefore, for each land use/cover type we calculated the percentage of “conversion loss to” or “conversion gain from”, in relation to the total “loss or gain” conversion of a land use/cover type according to equation (2) [22].

(2) $$\{\begin{array}{cc} P_{\text{loss}(i),j}=(p_{j,i}-p_{i.j})/(p_{i.}-p_{.i})\times 100 & i\neq j\\ P_{\text{gain}(i),j}=(p_{i,j}-p_{j.i})/(p_{i.}-p_{.i})\times 100 & i\neq j \end{array}$$

where:

*P_loss(i),j_* is the percentage taken by type *j* in the total “conversion loss” of category row *i*;
*P_gain(i),j_* is the percentage taken by type *j* in the total “conversion gain” of category row *i*;
*p_i,j_* and *p_j.i_* is the individual entry in a transition matrix A.

## 4. Results and Discussion

### 4.1. Land use/cover change

The land use maps for 1980, 1990 and 2000 are presented in Figure 2 and the transition matrices (Table 1 and 2) highlight the dominant dynamic events during the study period and reveal two distinct transition phases. From 1980 to 1990, although woodland and grassland increased by only 0.69% and 3.76%, the increased areas amount to 2,340 km^2^ and 13,330 km^2^. In contrast, cropland, water body, built-up land and unused land decreased by 1.69%, 17.63%, 35.91% and 14.25%, respectively, in the same period (Table 1). Among these changes, the most prominent was the conversion between woodland and grassland, and the next dominant change was between woodland and cropland. However, these trends reversed in the period from 1990 to 2000. The woodland and grassland decreased obviously by 0.97% and 3.33% and the decrease quantity amounts to 3,310 km^2^ and 12,260 km^2^. The trend of built-up land change was reversed significantly, increasing by 84.04%, from 1990 to 2000. Cropland, water body and unused land had expanded by 1.58%, 17.34% and 16.42%, respectively, in the same period (Table 2). Amount these changes, the most prominent one was the conversion from grassland to unused land at 17,244 km^2^. Although the transitions amount cropland, woodland and grassland still obvious, the retention frequencies during this period were much higher than the previous period. The percentage of the changed area was 26.7% from 1980 to 1990 and 10.7% from 1990 to 2000. To a large extent, the LUCC in the upper reaches of the Yangtze River from 1980 to 2000 was characterized by a severe replacement of cropland and woodland with grassland and built-up land.

The internal conversions between land use/cover types from 1980 to 2000 and the percentages taken by corresponding types in such loss or gain conversions were presented in Table 3. Over the period from 1980 to 1990, the increase of woodland and grassland accounted for 73.7% and 68.7% of the decrease of cropland, respectively. The reduction of water body and unused land was mainly due to the expansion of grassland in the same period. The decrease of built-up land during this period was caused basically by the regulations of national policies, which transferred a large amount of construction activities and investments from the west to the east and coastal part of China, leading to many infrastructures abandoned and converted to cropland and grassland. However, the expansion of woodland and grassland between 1980 and 1990 occurred mainly at the expense of cropland and unused land. It is worthy to note that although woodland increased obviously during this period, the internal conversion from woodland to grassland counteracted this trend mightily.

During the period from 1990 to 2000, the trend of internal structural variability of land use/cover in the upper reaches of the Yangtze River was almost opposite to the period before. The reduction of woodland and grassland was principally caused by the reclamation and degradation to unused land. By comparison, the increase of water body between 1990 and 2000 occurred mainly at the expense of unused land (60.8%) and cropland (20.6%). Although the expansion of built-up land may have taken up some part of previous woodland and grassland, most of these changes occurred on cropland, which accounted for 89.6% of the increase of built-up land from 1990 to 2000.

### 4.2. Changes of environmental factors related to LUCC

#### 4.2.1. Regional climate change

**Figure 3.**
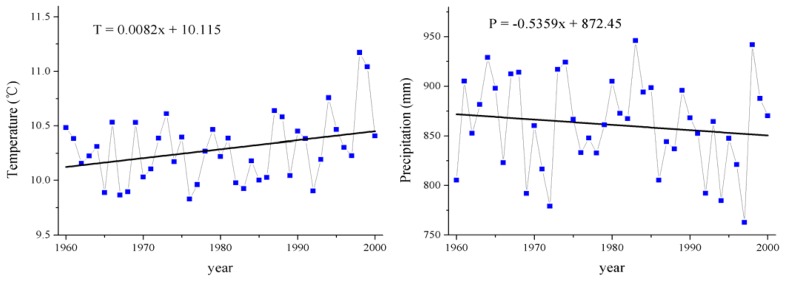
Climatic changes in the upper reaches of the Yangtze River during 1960-2000.

According to the analytical results of meteorological data recorded at 106 weather stations of the upper reaches of the Yangtze River and the adjacent region, since the 1960s the study area has exhibited a gradual warming trend with decreasing precipitation (Figure 3). The mean annual air temperature increased with a rate of 0.08°C per decade, and obviously increased with a higher rate during 1980 to 2000. The mean annual precipitation decreased with a rate of 5.4 mm per decade (Figure. 3). The relative study in the source region of the Yangtze River also showed a similar trend, where the air temperature has been rising since 1954 with a mean rising rate of 0.5°C per decade [46]. The annual precipitation in the source regions of the Yangtze River over the last 30 years showed no noticeable changes, or remained stable, but the summer annual precipitation (June-September) reduction reached 0.2-0.5 mm per year [46-47].

Temperature and precipitation are usually limiting factors affecting vegetation growth and agricultural development in the fragile ecosystem [18, 46-47]. Thus, the above-mentioned climatic changes were generally unfavorable to the expansion of cropland and high coverage grassland and also reduced the capability of soil water conservation. During the vigorous growth period, i.e. at the peak period for water requirement, the region's climate exhibited a tendency towards desiccation and hence the normal growth and reproduction of vegetation were seriously affected. What is particularly important is that such climatic change led to the significant alteration of the permafrost soil environment of the source region of the Yangtze River, which resulted in the decrease of soil moisture in the root zone, degradation of the high-cold meadow and swamp meadow vegetation and caused the succession of dominant plant species [46-47]. In addition, the increase of the mean annual temperature and the decrease of the mean annual precipitation likely contributed to the expansion of salinized land and sandy land and shrinkage of wetland and glacier, which are very harmful to environmental protection and sustainable agricultural development. Similar to the studies on Tibetan Plateau and highlands of Chiapas in Mexico, the land use dynamics also depended on other factors such as wind speed, evaporation and the availability of water sources [35, 48].

#### 4.2.2. Physical and ecological constraints

**Figure 4.**
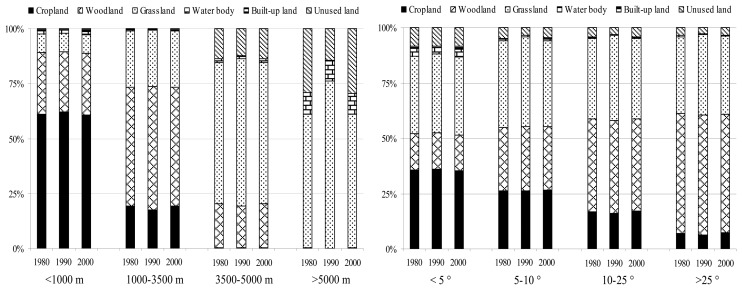
Land use/cover percentages in different elevation (left) and slope (right) ranges from 1980 to 2000. (Three columns in each elevation and slope range stand for the data of 1980, 1990 and 2000 from left to right).

The landform of the upper reaches of the Yangtze River is typically very irregular with about 40% of the area has the elevations of more than 3,500 m and 55% of the area having the inclinations of more than 10°. Figure 4 indicates the percentages of each land use/cover type in different elevation and slope ranges from 1980 to 2000. Within the <3,500 m elevation range, mostly cropland and built-up land were found, implying that population pressure caused the expansion of agricultural and built-up land into other areas with low elevations, while a substantial proportion of grassland and unused land were distributed in the >3,500 m elevation range, where the human intervention was relatively small. Similarly, most flat regions were used for crops and construction, with woodland and grassland interspersed, but a high proportion of crops were still cultivated in the inclinations ranging from 10-25° and even above 25°. Compared with agricultural activities on the plains, those steep agricultural fields could suffer from rapid soil erosion and nutrient depletion, which force farmers to abandon their plots after a few seasons of cultivation. Perhaps this may be one of the reasons for the abandonment or degradation of cropland during 1980-2000. There are some similar researches also from the hillside regions of Honduras and India where crop plots were still found at very high inclinations, even though there is severe risk of soil erosion and even landslides [49-50].

Although the area of cropland decreased by 283 km^2^ over the period from 1980 to 2000, most of which were occurred on lands with low elevations (<1,000 m) and inclinations (<5°). Within the higher elevation and inclination ranges, a trend of agricultural expansion was found during the same period (Figure 4). Forests degradation was mostly occurred in high elevation and slope areas, where were relatively inaccessible and received limited management. This could be one of the reasons for increased grassland in the same elevation and inclination ranges. However, the deforestation activities were mainly occurred in the <3,500 m elevation and <25° inclination ranges, which corroborates the results from other mountain areas [48-50]. This provides some indication that such deforestation occurred on a wider scale as a result of increasing human demand for living and economic benefit, especially in the developing countries. In addition, most of the built-up land and water body expansion between 1980 and 2000 occurred on lands with low elevations and inclinations, but water body decreased extensively in the higher elevation and inclination ranges, which supports the shrinkage of glacier on the mountains due to the increasing temperature.

Other studies such as Chen *et al.*, who examined slope degree and aspect, found strong impacts of slope on land use structure, but only a weak influence of aspect [16]. Hietel *et al.*, who investigated the relationships between land cover change and several physical variables (elevation, slope, soil texture and aspect), emphasized that greater diversity of physical landscape attributes is correlated with greater land cover dynamics [17]. Del Barrio *et al.* indicated the spatial heterogeneity of the landscape in mountain areas to be controlled by the physical environment [51]. Our study corroborates these results that physical attributes appear to be conditional factors of LUCC, especially in the mountain areas with great variation in topography.

### 4.3. Changes of socio-political factors related to LUCC

#### 4.3.1. Demographic and economic development

**Figure 5.**
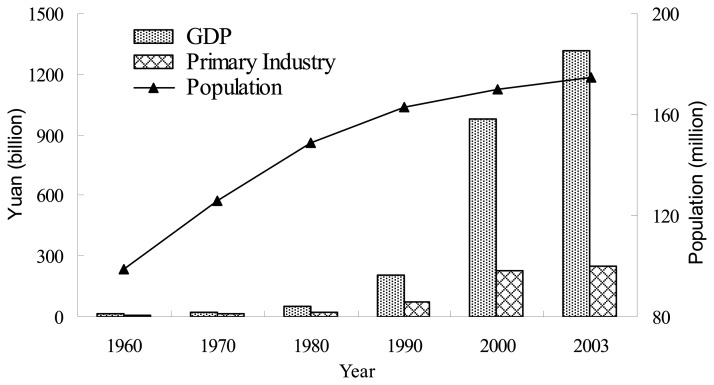
GDP and population changes in the upper reaches of the Yangtze River during 1960-2003.

Apart from environmental factors, demographic considerations and economic development also play important roles in the land use/cover dynamic of the upper reaches of the Yangtze River. Population growth has long been considered as a major factor leading to land use change [22, 52]. During 1980-2000, the population increased from 148.6 million to 169.9 million in the study area, with an estimated growth rate of 0.72% per year, which was much lower than that of the previous periods (Figure 5). This population increase was strongly correlated with economic developments in the same periods. The average annual growth rate of GDP and the primary industry in the study area were about 90.26% and 48.40% respectively between 1980 and 2000 (Figure 5).

It should be noted that since China initiated economic reforms and the so-called Open-Door Policy in 1978, economic development has increased greatly. In the upper reaches of the Yangtze River, a large proportion of the population remained in the agricultural sector and crop production and animal husbandry continued to play an important role in the local economies. To increase income and support a growing population, a large amount of cropland was converted to orchards, reservoirs and ponds or developed as industrial or urban land providing higher economic benefits. The remarkable increase of GDP during 1990 to 2000 was accompanied with a rapid expansion of built-up land and water bodies, which increased by 84.04% and 17.34%, respectively. Although the cropland losses promoted economic growth, the proportion of primary industry in the total GDP decreased gradually and the land utilization efficiency in the east and west parts of study area was quite asymmetrical. A similar study by Hietel *et al.* in Germany also indicated that the economic structure is an important factor influencing LUCC, as it relates to employment in the different economic sectors [10]. Obvious expansion of built-up land and evident shrinkage of native vegetation coverage resulting from improved socio-economic conditions and population growth also occurred in other areas in western China [35, 53-54].

#### 4.3.2. Institutions and natural resource policies

Land use/cover changes, while restricted by environmental conditions, are strongly driven by institutional and political factors. Government land management strategies tend to influence the decisions of individual farmers make on how natural resource are used. Land use is framed in an institutional and natural resources policy context, with property and land use rights, access to extension services and markets and agricultural price policies being additional relevant factors influencing these decisions [49-50]. The upper reaches of the Yangtze River is a special area with fragile ecosystem and getting immersed in many important policies, which bring about tremendous influences on its LUCC.

In the 1970s, because of many construction activities were transferred to the western China for the political and strategic reasons, a certain number of infrastructure and urban area have been constructed in the upper reaches of the Yangtze River and the configuration of land use changed significantly as well. However, after China initiated a series of economic reforms and the transition from a planned economy to a market-oriented economy since 1978, the developmental and economic activities of China were mostly concentrated in the eastern and coastal regions, leading to a certain extent abandonment of previous built-up areas in the study area during the early 1980s. The economic gap between eastern and western China and more employment opportunities in coastal cities induced the mass population migration from west to east, which in turn, led to many unproductive croplands abandoned due to labor shortage dealing with agriculture in our study area. In addition, under the Household Production Responsibility System, which was launched by China's government in 1980s, farms became more profit-oriented. As a result, a large proportion of agricultural lands have been withdrawn from crop cultivation for other production activities with higher income.

The loss of valuable cropland due to rapid urban sprawl during 1990 to 2000 has caught the attention of the central government [22, 32]. Since 1990, a large number of laws, regulations and decrees regarding land use, land protection and land acquisition have been promulgated by the State Council in China, such as the decree on Implementing the Land Management Law in 1991, the Regulations for the Protection of Basic Agricultural Land in 1994 and the Protection Rules of Basic Farmland in 1998. Although most of these laws and regulations focused on the protection of basic agricultural land and effectively slowed down the land conversion, some local governments implement the policies from the central government into local patterns of land use to get more benefits [11]. This usually results in the excessive loss of agricultural land and serious fragmentation of land use [22].

In order to rehabilitate the degraded eco-environmental situation and bring sandstorm, soil erosion and frequent flooding under control, the Chinese government initiated the construction of protective forest system in the middle-upper reaches of the Yangtze River from the late 1980s, and the Grain-for-Green Programme, also called the Conversion of Cropland to Forests and Grassland Programme in the late 1990s [11, 55-56]. In addition to these, at the turn of the century, the Western China Development Program, aiming to reorient the growth vigor towards the western region, and the construction of Three Gorges Dam also caused significant LUCC in the upper reaches of the Yangtze River [43, 57].

Although the importance of the socio-political factors in relation to LUCC is generally acknowledged [10-11, 18, 22, 31], the true effects from socio-political drivers are difficult to distinguish. This is mainly due to the multiple interactions and intercorrelations between land use/cover and environmental and socio-political variables [58] and the deficiency of available socio-political information with an appropriate spatial resolution [7]. However, the results from our study indicate that socio-political factors could prevail over environmental constrains on the extent and intensity of LUCC with the progress of technology and human society.

## 5. Conclusions

This paper analyzed the land use/cover dynamics and its environmental and socio-political forces in the upper reaches of the Yangtze River from 1980 to 2000 by using remote sensing, climatic and socio-economic data. The results of this study indicated that there had been significant land use/cover changes between 1980 and 2000 in the study area, which were characterized by a severe replacement of cropland and woodland with grassland and built-up land. The transition matrices highlight the dominant dynamic events and the internal conversions between land use/cover types during our study period and reveal two distinct transition phases. The percentage of the changed area was 26.7% from 1980 to 1990 and 10.7% from 1990 to 2000, indicating that an improved regulation and management strategies could enhance the retention of land resource.

Both environmental and socio-political factors are constraints to manage natural resource and play important roles in changing land use patterns [17, 50]. However, it is difficult to give a differential weight to the studied influencing parameters on current land use and their distribution. Our study indicates that the LUCC in the study area was evidently restricted by environmental attributes, such as climatic change and physical constraints. The slowly increasing temperature and the decreasing precipitation during 1960-2000 could restrict the expansion of cropland and high coverage grassland, reduced the capability of soil water conservation, and also likely in favor of the expansion of salinized land and sandy land and shrinkage of wetland. The correlation between physical constraints and LUCC presented in our study corroborates the findings of some earlier studies in mountainous areas [4, 49-50], that physical characters such as elevation and inclination could strongly affect the distribution of different land use types and agricultural activities and grazing should be controlled in areas with steep slopes.

Land use/cover changes in the upper reaches of the Yangtze River during 1980 to 2000, while restricted by environmental attributes, were strongly driven by socio-political factors. The gradually increases of population and economic development have caused markedly changes of land use, which characterized by a large amount of cropland were converted to orchard, reservoir and pond and developed as industrial or urban land with higher economic benefits. In addition, the upper reaches of the Yangtze River is a special area getting immersed in many important policies, which bring about tremendous influences on its land use/cover change.

However, excessively pursuing higher land use benefits likely results in seriously environmental degradation. Restructuring of land use should be based on land suitability and sustainable development, rather than only based on the short-term economic benefits of land use. Therefore, our study suggests that more effective planning and policies will be required to manage urban growth and protect basic cropland. The concept of an urban growth boundary and incentive-based policies such as development impact fees and location efficient mortgages may be helpful for local government to stem the tide of urban sprawl [22, 59-60]. In addition, soil water conservation, appropriate agricultural technologies, and alternate energy source to fire wood are important for development in our study area. The government should promulgate accessorial regulations and policies for carrying out the Grain-for-Green Programme, since only after improvement of the socio-economic situation can sloping cropland be converted to woodland or grassland in the long run [43]. Furthermore, a multidisciplinary collaboration among the various government departments, non-governmental organizations and academia could improve the planning and implementation of management strategies.

A thorough comprehension of historical land use/cover helps to explain current LUCC and to understand ecological structures, processes and functions based on it [10]. This study may contribute to both the ecologist and the manager working at the regional level. Whereas the former will benefit from the understanding of pattern dynamics which might affect ecological processes in landscape scale, the latter will be better equipped to develop effective management strategies and policies for the rational land use and environmental protection in the upper reaches of the Yangtze River. Aside from these findings, some other important concerns that need to be addressed by future researches could be the evaluation of ecological consequences of these LUCC and how to forecast areas potentially sensitive to future changes.

## Figures and Tables

**Table 1.** Transition matrix of each land use type in 1980 and 1990, and its change in 1990 (10^3^ km^2^).

Land use/cover type in 1990			Land use/cover type in 1980					Changes in 1990 (%)

	CL	WL	GL	WB	BUL	UL	Total	
CL	**157.119**	35.576	19.095	1.856	2.375	0.062	216.083	-1.69
WL	38.309	**255.174**	43.947	0.57	0.26	1.104	339.364	0.69
GL	21.642	44.799	**273.515**	3.862	0.313	24.031	368.162	3.76
WB	1.523	0.472	2.693	**6.452**	0.123	0.669	11.932	-17.63
BUL	1.149	0.171	0.123	0.101	**1.175**	0.006	2.725	-35.91
UL	0.048	0.831	15.461	1.645	0.006	**29.427**	47.418	-14.25
Total	219.79	337.023	354.834	14.486	4.252	55.299	985.684	

Note: CL = Cropland, WL = Woodland, GL = Grassland, WB = Water body, BUL = Built-up land, UL = Unused land; the unchanged area of each land use/cover type was marked in bold.

**Table 2.** Transition matrix of each land use type in 1990 and 2000, and its change in 2000 (10^3^ km^2^).

Landuse/covertype in 2000	Land use/cover type in 1990							Changesin 2000(%)

	CL	WL	GL	WB	BUL	UL	Total	
CL	**195.262**	15.825	7.99	0.191	0.223	0.016	219.507	1.58
WL	11.612	**307.774**	16.209	0.083	0.035	0.344	336.057	-0.97
GL	6.3	14.974	**325.857**	0.432	0.031	8.308	355.901	-3.33
WB	0.618	0.194	0.698	**11.07**	0.022	1.4	14.001	17.34
BUL	2.275	0.148	0.164	0.015	**2.412**	0.001	5.015	84.04
UL	0.018	0.45	17.244	0.142	0.002	**37.349**	55.204	16.42
Total	216.083	339.364	368.162	11.932	2.725	47.418	985.684	

Note: CL = Cropland, WL = Woodland, GL = Grassland, WB = Water body, BUL = Built-up land, UL = Unused land; the unchanged area of each land use/cover type was marked in bold.

**Table 3.** The percentages taken by corresponding types in internal conversions among land use types.

Corresponding land use/cover type	Land use/cover type (1980-1990)						Land use/cover type (1990-2000)					
	
	CL^−^	WL^+^	GL^+^	WB^−^	BUL^−^	UL^−^	CL^+^	WL^−^	GL^−^	WB^+^	BUL^+^	UL^+^
CL		116.7	19.1	13.0	80.3	0.2		127.5	13.8	20.6	89.6	0
WL	73.7		6.4	3.8	5.8	3.5	122.9		10.1	5.4	4.9	1.4
GL	68.7	-36.4		45.8	12.4	108.7	49.4	-37.4		12.9	5.8	114.8
WB	-9.0	4.2	8.8		1.4	-12.4	-12.4	3.3	2.2		-0.3	-16.2
BUL	-33.1	3.8	1.4	-0.9		0	-59.9	3.4	1.1	0.3		0
UL	-0.4	11.7	64.3	38.2	0		-0.1	3.2	72.8	60.8	0	

Note: CL = Cropland, WL = Woodland, GL = Grassland, WB = Water body, BUL = Built-up land, UL = Unused land; - conversion loss to, + conversion gain from.
